# Identification of Bulgarian Sourdough Microbiota by Metagenomic Approach Using Three Commercially Available DNA Extraction Protocols

**DOI:** 10.17113/ftb.61.01.23.7796

**Published:** 2023-03

**Authors:** Ivelina Vassileva, Vesselin Baev, Galina Yahubyan, Elena Apostolova-Kuzova, Angel Angelov, Miglena Koprinarova

**Affiliations:** 1 Institute of Molecular Biology “Acad. Roumen Tsanev”, Bulgarian Academy of Sciences, Acad. G. Bonchev Str. bl. 21, 1113 Sofia, Bulgaria; 2 Department of Plant Physiology and Molecular Biology, Tzar Assen 24, University of Plovdiv,; 4000 Plovdiv, Bulgaria; 3 Department of Biotechnology, University of Food Technologies, 26 Maritza Blvd., 4002 Plovdiv, Bulgaria; 4 Department of Catering and Nutrition, University of Food Technologies, 26 Maritza Blvd., 4002 Plovdiv, Bulgaria

**Keywords:** sourdough, DNA extraction methods, V1-V3 16S rRNA, next-generation sequencing, metagenomics, microbiota

## Abstract

**Research background:**

Sourdough is a spontaneously formed, complex microbial ecosystem of various lactic acid bacteria (LAB) and yeast which, by producing specific metabolites, determines the quality of the baked products. In order to design and control the sourdough with preferred nutritional characteristics, it is crucial that the LAB diversity of the product of interest be elucidated.

**Experimental approach:**

Using the opportunities of next-generation sequencing (NGS) of the V1-V3 hypervariable gene region of 16S rRNA, we studied the microbial ecosystem of a whole grain sourdough made of *Triticum monococcum*, originating from Southwestern Bulgaria. Since the DNA extraction method is considered crucial for the accuracy of the sequencing results, as it can introduce significant differences in the examined microbiota, we used three different commercial kits for DNA isolation and analyzed their impact on the observed bacterial diversity.

**Results and conclusions:**

All three DNA extraction kits provided bacterial DNA which passed quality control and was successfully sequenced on Illumina MiSeq platform. The results received from the different DNA protocols showed variations in the microbial profiles. Alpha diversity indices (ACE, Chao1, Shannon, and Simpson) were also different among the three groups of results. Nevertheless, a strong dominance of phylum Firmicutes, class Bacilli, order Lactobacillales, represented mostly by family Lactobacillaceae, genus *Lactobacillus* (relative abundance of 63.11–82.28%) and family Leuconostocaceae, genus *Weissella* (relative abundance of 3.67–36.31%) was observed. *Lactiplantibacillus plantarum* and *Levilactobacillus brevis* with relative abundance of 16.15–31.24% and 6.21−16.29% respectively, were the two dominant species identified in all three DNA isolates.

**Novelty and scientific contribution:**

The presented results give insight into the taxonomic composition of bacterial community of a specific Bulgarian sourdough. Having in mind that the sourdough is a difficult matrix for DNA isolation on the one hand, and that there is no standardized DNA extraction protocol for this matrix on the other hand, this pilot study aims to give a small contribution to the future establishment and validation of such a protocol, which will allow accurate assessment of the specific microbiota of sourdough samples.

## INTRODUCTION

In the last few years, the interest in the products made of whole grain flour by sourdough fermentation has been growing fast in European markets. The use of sourdough improves the flavour, structure and stability of baked goods and ameliorates the nutritional qualities of the whole grain products by delaying flour digestibility, thus decreasing glycaemic response, increasing protein digestibility and improving the bioavailability of mineral substances. It is suggested that the increased intake of sourdough products helps to improve conditions like diabetes, cardiovascular and intestinal diseases (*1*). Whole grain flour is known to contain much more vitamins, minerals, fibre, antioxidants, carotenoids, flavonoids and phenolic acids than the refined wheat flour, and sourdough fermentation increases the amount of beneficial microorganisms in whole wheat products, which improves human health (*1*). Sourdough has a dynamic ecosystem of fermentation organisms. The most common LAB species are *Fructilactobacillus sanfranciscensis* (former *Lactobacillus sanfranciscensis*), *Lactiplantibacillus plantarum* (former *Lactobacillus plantarum*), *Levilactobacillus brevis* (former *Lactobacillus brevis*), *Pediococcus pentosaceus*, *Companilactobacillus alimentarius* (former *Lactobacillus alimentarius*), *Limosilactobacillus pontis* (former *Lactobacillus pontis*), *Furfurilactobacillus rossiae* (former *Lactobacillus rossiae*) (*2*-*4*), some species of *Leuconostoc* and *Weissella*, as well as yeasts *Saccharomyces cerevisiae* and species of the *Kazachstania* clade (*5*-*7*). The qualitative characteristics of the fermented sourdough are defined by LAB diversity and yeast flora in it (*8*-*11*). The different ingredients, environmental factors and fermentation conditions define the uniqueness of each sourdough starter, characterized by a complex microbial ecosystem of interacting yeast and bacteria. Better understanding of the factors affecting sourdough microbiota provides opportunity for selection of starter cultures and fermentation conditions, which can improve the sourdough breadmaking process and the production of baked goods with desirable quality. Hence, our research was focused on the investigation of bacterial microbiota in traditional Bulgarian whole grain sourdough made of *Triticum monococcum* flour by means of next-generation sequencing (NGS) technology.

One of the most important methods of conventional microbiome analysis of food is 16S DNA sequencing, which provides insight into the microbial ecosystem of a product with a precise taxonomic resolution. The 16S rRNA gene contains nine hypervariable regions flanked by conserved sequences, which allow amplification and sequencing of target regions and taxonomic identification of the food-associated bacterial species. The sequences are clustered into operational taxonomic units (OTUs) which are then compared against databases for identifications of the bacteria present in the microbiome. Recently, new methods have been developed that resolve amplicon sequence variants (ASVs) from NGS amplicon data without disadvantages that define molecular OTUs (*12*-*17*). ASV methods infer the biological sequences in the sample prior to the introduction of amplification and sequencing errors, and discriminate sequence variants differing by as little as one nucleotide.

NGS techniques are very efficient for studying the food microbiota, but there are some methodological features crucial for the success of the sequencing. One critical step affecting the accuracy of 16S rRNA NGS data is the DNA extraction. The isolation of a good quantity and quality DNA depends on the protocol used for a particular matrix (*18*) and affects the experimental results. That is why it is essential that an optimal extraction method should be used (*19*-*23*). A recommended step in DNA isolation from food matrices is the removal of background DNA of plant or animal origin that would affect the relevant sequence information. The matrix contents may also interfere with the performance of molecular analysis as it may inhibit the required biochemical reactions (*22*). A potential approach to eliminate matrix components is to retrieve microbes by centrifugation (*24*) or differential centrifugation and filtration from aqueous solutions (*25*). Concerning the sourdough, the elasticity of the samples further complicates the analysis, which requires thorough homogenization of the samples.

Different commercial kits have been used for DNA extraction for metagenome analysis from sourdough: DNeasy Blood and Tissue kit (Qiagen, Hilden, Germany) (*26*), DNeasy PowerSoil kit (Qiagen, Hilden, formerly MoBio) (*27*) as well as from other matrices: Zymo Quick-DNA Fecal/Soil Microbe Mini-prep kit (Ozyme, Saint-Cyr-l’Ecole, France) (*28*), DNeasy Blood and Tissue kit (Qiagen, Venlo, the Netherlands) (*29*). However, the choice of DNA extraction kit for the isolation of bacterial DNA from sourdough is still difficult and further investigation is required to elucidate the efficiency of the commercial kits available on the market.

In this research, we chose V1-V3 hypervariable region of 16S rRNA for NGS and analysed the microbiota of Bulgarian sourdough from *T. monococcum*. For better characterization, we used three different DNA extraction kits from three manufacturers namely: NucleoSpin Food kit (Macherey-Nagel, Düren, Germany) for DNA extraction from food samples specifically, QIAamp DNA Microbiome kit (Qiagen, Hilden, Germany) for the isolation of bacterial microbiome DNA, and GENESpin kit (Eurofins Technologies, Budapest, Hungary) for the isolation of high-quality DNA from food and feed samples. Two of the kits (NucleoSpin and GENESpin) are designed to extract DNA from food, while QIAamp is intended for depletion of host DNA and extraction of microbiome DNA from mixed samples. In addition, NucleoSpin and QIAamp kits were chosen because they have already been used in published metagenomic analyses of food (*30*) and other type of samples (*31*). To the best of our knowledge, the GENESpin kit has not been applied for metagenomic analysis so far.

## MATERIALS AND METHODS

### Sourdough

**Fig. S1:**
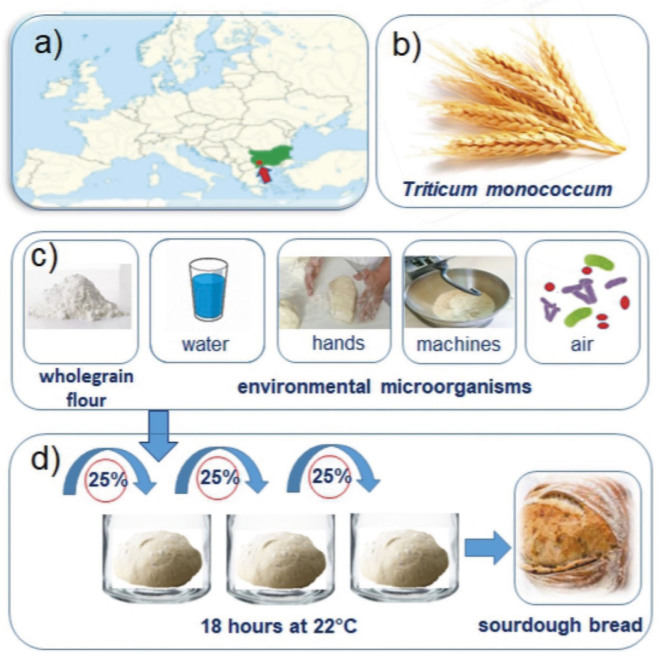
Schematic representation of geographical, plant origin and production workflow of the investigated Bulgarian sourdough: a) the sourdough was made by a manufacturer located in Southwestern Bulgaria (town of Bansko, red dot), b) the type of flour used was *Triticum monococcum*, c) ingredients of the sourdough and sources of microorganisms, d) overview of the production workflow process. Fermentation of the sourdough proceeded 18 h at 22 °C and included three backsloppings. For each backslopping, 1.5% NaCl was added and 25% of the sourdough was used

The sourdough originated from a manufacturer of typical Bulgarian bread based in Southwestern Bulgaria (Fig. S1). The whole grain flour used was from *Triticum monococcum*. The technological time for the fermentation of the sourdough was 18 h at 22 °C and included three backsloppings. For each backslopping, 1.5% NaCl (*m*/*V*) was added and 25% of the sourdough was used. The sample was taken after the last backslopping and was kept and transported frozen until the DNA extraction was performed.

### DNA extraction

We compared three kits to evaluate their capability to extract high quality DNA from sourdough for 16S rRNA NGS analysis (Table 1). These are namely NucleoSpin Food kit (Macherey-Nagel, Düeren Germany) for DNA extraction from food samples specifically, QIAamp DNA Microbiome Kit (Qiagen, Hilden, Germany) for isolation of bacterial microbiome DNA form mixed samples, and GENESpin kit (Eurofins Technologies) for isolation of high-quality DNA from food and feed samples. These three different commercial kits were used for DNA extraction from sourdough according to the manufactures’ protocols with several optimizations for the particular matrix. The names of all reagents (*e.g.* buffers and enzymes) mentioned hereafter are as provided by the respective manufacturer.

**Table 1:** Main characteristics of the used DNA extraction kits

DNA extraction kit	Removal of background DNA	Cell disruption			DNA purification	*V*(elution buffer)/µL	Cost per sample/€
Thermal	Chemical	Mechanical
NucleoSpin Food kit (Macherey-Nagel, Germany)	no	65 °C	yes	no	NucleoSpin Food column	100 Buffer CE	4.02
QIAamp DNA Microbiome kit (Qiagen, Germany)	yes	56 °C	yes	yes TissueLyser	QIAampUCP Mini column	30 Buffer AVE	11.92
GENESpin Food kit (Eurofins Technologies, Hungary)	no	65 °C	yes	no	GENESpin column	100 GENESpin buffer	2.98

### Method 1 (NucleoSpin Food kit)

DNA isolation with NucleoSpin Food kit was performed in the laboratories of Eurofins Genomics Europe Sequencing GmbH, Konstanz, Germany. The protocol had been previously validated. DNA was extracted from 200 mg of the starting sample material after homogenization. The NucleoSpin Food protocol consists of six steps for the extraction of genomic DNA (plant, animal or bacteria) from food samples. It guarantees good recovery of small genomic DNA fragments (<1 kbp) out of complex food matrices. The sample was first well homogenized. Then, 550 μL of lysis buffer CF and 10 μL proteinase K (10 mg/mL) were added to the homogenate and incubated for 30 min at 65 °C. After centrifugation for 10 min at 10 000×*g*, to the clear supernatant the same volumes of buffer C4 and ethanol were added. For DNA binding, the sample was loaded (max. 700 μL at a time) onto the NucleoSpin Food column, which was placed in a collection tube and centrifuged for 1 min at 11 000×*g*. After discarding the flow-through, the remaining sample was loaded. To wash the silica membrane, 400 μL of buffer CQW were added, the column was centrifuged for 1 min at 11 000×*g* and the flow-through was discarded. Afterwards, 700 μL of buffer C5 were added onto the NucleoSpin column and centrifuged for 1 min at 11 000×*g*. For the third wash, 200 μL of buffer C5 were pipetted onto the column and centrifuged for 2 min at 11 000×*g* in order to remove residual ethanol completely. To elute the DNA from the membrane, the column was placed in a new 1.5-mL microcentrifuge tube and 100 μL of elution buffer CE preheated to 70 °C were added. After 5 min of incubation at room temperature, it was centrifuged for 1 min at 11 000×*g* to elute the DNA.

#### Method 2 (QIAamp DNA Microbiome kit)

The QIAamp DNA Microbiome kit provides selective isolation of bacterial DNA from mixed samples. The protocol consists of six steps, where the first two steps assure differential lysis and degradation of background (plant) DNA while keeping the bacterial cells intact. The nature of the test samples required pre-suspension, which was performed in phosphate-buffered saline (PBS, 1.8 mM KH_2_PO_4_, 10.0 mM Na_2_HPO_4_, 2.7 mM KCl, 137.0 mM NaCl, pH=7.5) buffer, pH=7.5 (*32*) at a ratio of 1:1 (*m*/*V*). To remove coarse particles, the suspension was centrifuged at 2000×*g* for 1 min (centrifuge model 5418; Eppendorf AG, Hamburg, Germany). Aliquots of each sample were used for two parallel extractions. For each extraction, 500 μL buffer AHL were pipetted to 1 mL of the suspension in a 2-mL tube (Eppendorf). The mixture was incubated for 30 min at room temperature with rotation and then centrifuged at 10 000×*g* for 10 min. After removing the supernatant, 190 μL of buffer RDD (QIAamp DNA Microbiome kit) and 2.5 μL of benzonase were pipetted and mixed well prior to incubation at 37 °C for 30 min at 600 rpm in a heating block (Thermomixer Comfort, Eppendorf AG). Following the treatment with 20 μL proteinase K at 56 °C for 30 min at 600 rpm, and briefly spinning the tube at slow speed, 200 μL of buffer ATL (with reagent DX) were mixed well with the sample to avoid loss of sample material. With this final step, the lysis of the eukaryotic cells was completed. The third step of chemical and mechanical disruption of bacterial cells included transfer of the sample into a pathogen lysis tube L for cell lysis in a tissue lyser LT, FastPrep24 instrument (Qiagen, Hilden, Germany) for disruption (twice at 50 Hz for 10 min). After centrifugation of the pathogen lysis tube L, 40 μL proteinase K were added to the supernatant and 200 μL of the buffer APL2 were added by pulse vortexing. After incubation, 200 μL ethanol were added to the lysate. To remove the intact non-lysed cells, the lysate was centrifuged at 2000×*g* for 1 min. The fourth step was the lysate adsorption to the QIAampUCP membrane. At this step, the lysates from the two parallel extractions were combined (approx. final volume 1600 μL) and 700 μL were transferred into the QIAampUCP Mini column (QIAamp DNA Microbiome kit) followed by centrifugation at 6000×*g* for 1 min. The rest of the lysate was loaded on the same column and centrifuged under the same conditions. The next two steps included washing with 500 μL of buffer AW1, followed by centrifugation and then the addition of buffer AW2 and centrifugation at full speed for 3 min. The QIAampUCP Mini Column was placed into a fresh 2-mL collection tube and centrifuged at full speed for 1 min. The last step of DNA elution was performed by adding 30 μL buffer AVE onto the column, followed by 5 min incubation at room temperature and centrifugation at 6000×*g* for 1 min.

#### Method 3 (GENESpin kit)

The GENESpin kit is designed for the isolation of genomic DNA from food and feed samples of plant and animal origin. The kit assures good recovery rates for small genomic DNA fragments (<1 kbp). This protocol consists of six steps including three washing steps, like the NucleoSpin Food protocol. The mass of starting sample material was 200 mg, taken after previous homogenization. The second step for cell lysis included adding 550 μL of GENESpin lysis buffer, preheated to 65 °C (water bath WNB14; Memmert, Schwabach, Germany), and incubation with 10 μL proteinase K (10 mg/mL, GENESpin Kit) for 30 min at 65 °C. A volume of 10 μL RNAse A (20 mg/mL, Eurofins Technologies) was added to the sample and incubation for 30 min at room temperature was performed. Following centrifugation (centrifuge model 5418; Eppendorf AG), the supernatant was transferred into a new centrifuge tube (Eppendorf AG), then the same volumes of GENESpin binding buffer and ethanol (99.9%; Merck, Darmstadt, Germany) were added to the sample. The fourth step was binding of the DNA onto the column matrices. A GENESpin column was placed into a new collection tube (Eppendorf AG) and 700 μL of the sample were loaded onto the column. Afterwards, the sample was centrifuged, the flow-through was discarded and this step was repeated. The fifth step was washing the sample with three washing substeps. First, 400 μL of GENESpin wash buffer 1 were added to the column and centrifuged, then 700 μL of GENESpin wash buffer 2 were added and centrifuged, and the final 200 μL volume of GENESpin wash buffer 2 were added and centrifuged for 2 min. The last step included loading 100 μL GENESpin elution buffer onto the membrane, 5-minute incubation and 1-minute centrifugation at 11 000×*g* to elute the DNA.

For all three extracted DNA isolates, PCR and quality control were performed by Eurofins Genomics in order to check whether a PCR product could be generated. Based on this quality control, which met the requirements for the amount, concentration and quality of the extracted DNA, all three isolates were subjected to next-generation sequencing.

### DNA quantification

DNA quantity was determined fluorometrically with Qubit 4 fluorometer (Invitrogen™, Thermo Fisher Scientific, Waltham, MA, USA). DNA integrity was checked by electrophoresis on 1% agarose gel.

### Next-generation sequencing of 16S rRNA gene amplicons

The amplification of target sequences of 16S rRNA gene and NGS was performed by Eurofins Genomics. The extracted DNA was subjected to PCR amplification of 16S rRNA gene sequences from the hypervariable regions V1-V3 for bacterial profiling. The amplicons were generated from the samples *via* two-step PCR protocol. PCR products were subjected to sequencing on Illumina MiSeq platform 2×300 bp paired-end reads.

### Bioinformatic data analysis

We used Cutadapt v. 1.9 program (*15*) for the process of quality filtering and trimming of the pair-end reads. Metagenomics analysis was done using the DADA2 (v. 1.14) pipeline as described by Callahan *et al.* (*33*), which was recommended to replace OTU with amplicon sequence variant (ASV)-based approaches (*34*). Quality checks were conducted, clean reads were denoised, dereplicated and filtered for chimeras to generate ASVs. The resulting ASV table was used for all downstream analyses, including taxonomic assignment, phylogenetic alpha diversity measurements, differential abundance comparisons, and visualizations. Taxonomic assignment of sequence variants was performed using a combination of the functions assignTaxonomy and assignSpecies and was compared using the SILVA reference database (v. 132) (*35*). We generated within-sample microbial diversity (alpha diversity) indices (ACE, Chao1, Shannon and Simpson) by using QIIME package (alpha_diversity.py -o alphadiv --metrics 'ace,chao1,observed_otus,shannon,simpson') (*36*). Krona charts for visualization are created from the samples in ASV table (*37*).

## RESULTS AND DISCUSSION

### DNA extraction and quantification

**Fig. 1:**
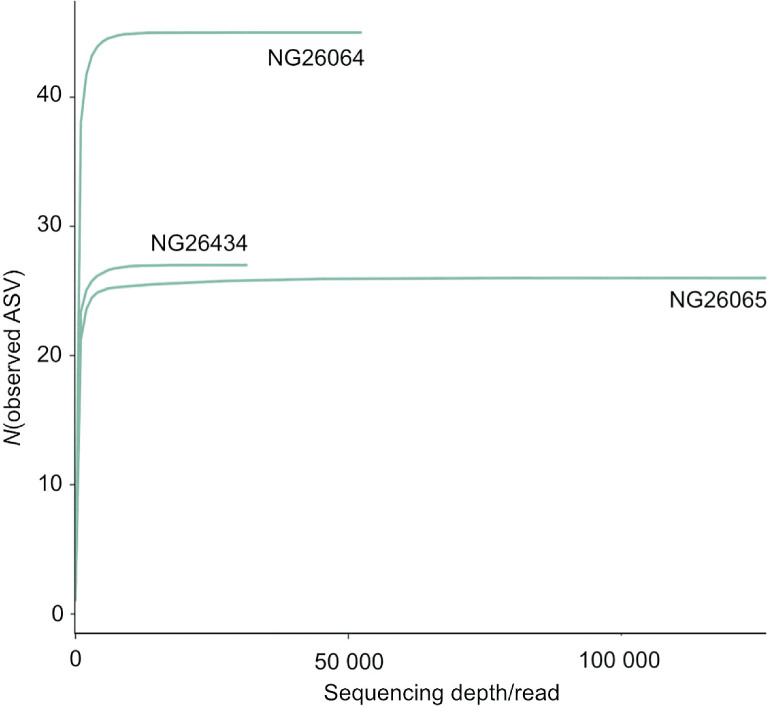
Rarefaction curves constructed by using amplicon sequence variants (ASVs) observed in the three DNA isolates (NG-26064, NG-26065 and NG 26434). On the abscissa are plotted the reads sequenced from the DNA extracted with NucleoSpin Food kit (NG-26064), QIAamp DNA Microbiome kit (NG-26065) and GENESpin Food kit (NG-26434). On the ordinate is shown the number of found ASVs. Each curve represents a particular isolate

In this research, we investigated the microbiota of spontaneously fermented sourdough from *Triticum monococcum*, originating from Southwestern Bulgaria, by 16S rRNA gene next-generation sequencing (NGS). In order to do that, we used three commercial kits for DNA extraction (Table 1). The quantity and yield of the extracted DNA are presented in Table 2. The concentrations of the extracted DNA ranged from 29.2 to 246.8 ng/µL, while the total DNA yield was between 0.88 and 24.68 µg depending on the DNA extraction method. The differences in the DNA concentration and yield could be partly explained with the methodological features of the protocols. GENESpin as well as NucleoSpin kit do not include a step for depletion of background DNA, so the DNA obtained with these two kits also included DNA with plant origin. With the QIAamp kit, the DNA with eukaryotic origin was significantly reduced (see below). There are recommendations in the literature for reduction of host (background) DNA in the isolates for microbiome analysis (*31*). Using the same QIAamp DNA Microbiome kit among others, Bjerre *et al.* (*31*) advised reduction of host DNA before 16S metagenomic analysis and proved that the protocol did not introduce taxonomic biases. Although the QIAamp kit provided selective isolation of bacterial DNA, one drawback of using this kit was the hands-on time, which in our case was 50 min longer than with the other two kits, due to the additional step for degradation of plant cells. Moreover, the price of this kit was higher (€11.92 per sample) than the prices of the other two kits (€4.02 for NucleoSpin and €2.98 for GENESpin) (Table 1). Importantly, despite the differences, all three protocols (Method 1, Method 2 and Method 3, see Materials and Methods) provided DNA with quality and quantity that met the requirements of the NGS quality control. Our results confirmed previously published data which showed that the success of the 16S rRNA gene sequencing is generally independent of the concentrations of DNA in the samples (*31*, *38*). Accordingly, with the QIAamp kit we obtained a lower DNA yield (Table 2), which was expected considering that this kit, unlike the other two, eliminates plant DNA, which is much more strongly represented in the sample than the bacterial DNA. However, with the same QIAamp isolate, we achieved the highest number of reads (Table 3) and significantly increased sequencing depth (Fig. 1).

**Table 2:** Summary of the concentration and yield of DNA extracted from sourdough with three commercial kits

Extraction method	Isolate	*m*(sample)/mg	γ(DNA)/(ng/µL)	*γ*(DNA)_total_/µg
NucleoSpin Food kit (Macherey-Nagel, Germany)	NG-26064	200	246.8	24.68
QIAamp DNA Microbiome kit (Qiagen, Germany) GENESpin Food kit (Eurofins Technologies, Hungary)	NG-26065 NG-26434	1000 200	29.2 210.1	0.88 21.01

**Table 3:** Alpha diversity indices (ACE, Chao1, Shannon and Simpson) of the 16S rRNA gene libraries of the sourdough from MiSeq sequencing analysis

Isolate	Read	ASV	ACE	Chao1	Shannon	Simpson
NG-26064	52474	45	45	45	4.26	0.92
NG-26065	126553	26	26	26	2.70	0.77
NG-26434	31528	27	27	27	3.78	0.91

ASV=amplicon sequence variant

### Metagenomic data analysis

We chose the V1-V3 region of 16S rRNA gene for sequencing. In total, 210 555 reads were analysed from the three DNA isolates of the sourdough. The number of reads in NG-26064 isolate (Method 1) was 52474, in NG-260665 isolate (Method 2) was 126 553, and in NG-26434 isolate (Method 3) 31 528 (Table 3). The observed richness, presented by identified amplicon sequence variants (ASVs), was reported as follows for the particular DNA isolates: 45 ASVs for NG-26064, 26 ASVs for NG-26065, and 27 ASVs for NG-26434. The total number of ASVs found was 98 (Table 3). In agreement with another report (*38*), our results showed that the DNA isolation method might influence the number of the observed ASVs.

The effect of the extraction methods on alpha diversity was assessed by Chao1, ACE, Shannon and Simpson indices. For evaluation of the species richness, ACE and Chao1 indices were estimated (*39*). Lower values for both indices were calculated for NG-26065 (QIAamp) and NG-26434 (GENESpin) isolates. Higher richness indices were calculated for NG-26064 (NucleoSpin) isolate (Table 3). The diversity indices Shannon and Simpson (*39*) were also calculated for each isolate. The lowest diversity was obtained with the QIAamp Kit, intermediate diversity with the GENESpin Kit, whereas the highest diversity was achieved again by the NucleoSpin Kit. Therefore, the NucleoSpin Kit differs from the other two kits with higher Chao1/ACE richness and Shannon/Simpson diversity. This correlated with the higher number of ASVs in this isolate (Table 3). In agreement with the data from the literature, our results showed that the different DNA extraction methods (Table 1) might influence the bacterial species richness and diversity (*40*).

We generated rarefaction plot to investigate whether the sequencing depth was sufficient to assess the microbiota in all three isolates. The calculated sequencing depth was based on alpha diversity analyses (Fig. 1). Of the three observed rarefaction curves each reached a plateau, suggesting that the ASVs presented in all isolates were almost completely determined and a potential increase of the number of reads would not significantly contribute to the number of ASVs. QIAamp isolate had the highest sequencing depth, whereas GENESpin isolate had the lowest. The comparative analysis highlighted NucleoSpin as the best among the three kits for the extraction of bacterial DNA from sourdough for NGS, since at sufficient sequencing depth the number of ASVs was much bigger than of those achieved with the other two kits. Nevertheless, the rarefaction curve analysis showed that the depth of 16S rRNA gene sequencing was adequate in all three isolates, which allowed observation of the bacterial community of the sourdough.

### Taxonomic profile of the sourdough sample

In agreement with data from the literature (*41*), the 16S rRNA sequence analysis revealed that Gram-positive bacteria were dominant in the investigated sourdough compared with Gram-negative. The dominant taxon at phylum level was Firmicutes (87.67%), established at 86% in NG-26064 isolate, 100% in NG-26065 and 77% in NG-26434 isolate. Bacilli were the most dominant class, 86% in NG-26064 isolate, 100% in NG-26065 isolate and 77% in NG-26434 isolate. Lactobacillales were found as the most dominant order affiliated with class Bacilli, present in 86% in NG-26064 isolate, 100% in NG-26065 isolate, and 77% in NG-26434 isolate. At the family level, Lactobacillaceae were found as dominant, represented as follows: 82% in NG-26064, 63% in NG-26065 and 70% in NG-26434. The sub-dominant family found was Leuconostocaceae, represented at 4% in NG-26064, 36% in NG-26065 and 7% in NG-26434 GENESpin (see also Supplementary data).

**Fig. 2:**
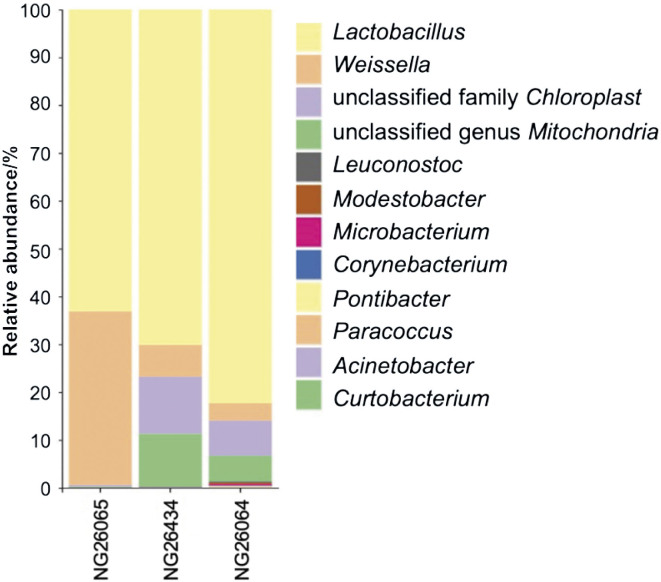
Relative abundance and taxonomic assignment of bacterial community at the genus taxonomic level of the three sourdough isolates. The colour key on the right shows identified genera

The NGS analysis revealed the following ten genera in the analyzed sourdough: *Lactobacillus, Weissella, Modestobacter, Microbacterium, Leuconostoc, Corynebacterium, Pontibacter, Paracoccus, Curtobacterium* and *Acinetobacter* (Fig. 2). Among them, *Lactobacillus* was the dominant genus, at 82.28% in NG-26064 isolate, 63.11% in NG-26065 isolate and 70.08% in NG-26434 isolate (Fig. 2). It is important to clarify that in the present work, due to the data obtained from the software (DADA2), we used the old classification of genus *Lactobacillus,* before its reclassification into 25 genera and the union of Lactobacillaceae and Leuconostocaceae published by Zheng *et al.* (*42*) in 2020. *Weissella* was the sub-dominant genus in all three isolates, represented at 3.67% in the DNA extracted with NucleoSpin, at 36.31% in QIAamp isolate, and at 6.69% in GENESpin isolate (Fig. 2). The other genera were detected at very low abundance, less than 0.5%. Having in mind that DADA2 software provides very restrictive data from the 16S rRNA gene NGS analysis, we considered that although the percentages are low, the presence of these genera in the DNA isolates is important to note. Accordingly, the genus *Leuconostoc* was identified in the three isolates, in NG-26064 at 0.24%, NG-26065 at 0.09% and in NG-26434 at 0.18%. Genus *Acinetobacter* was represented at 0.04% in NG-26064 and at 0.06% in NG-26434. We did not detect this genus by means of QIAamp kit (NG-26065). The other abovementioned genera were found only in NG-26064 isolate as follows: *Modestobacter* 0.30%, *Microbacterium* 0.29%, *Corynebacterium* 0.13%, *Pontibacter* 0.13%, *Paracoccus* 0.12% and *Curtobacterium* 0.06%.

In agreement with the data from the literature (*43*), facultative heterofermentative *Lactiplantibacillus plantarum* was found in codominance with obligately heterofermentative LAB in the investigated sourdough. *L. plantarum* was present at 16.15% in NG-26064 (NucleoSpin), at 16.28% in NG-26434 (GENESpin) and at 31.24% in NG-26065 (QIAamp). Obligate heterofermentative *Levilactobacillus brevis* was found with relative abundance of 14.46 and 16.29% in NG-26064 and NG-26434, respectively, and 6.21% in NG-26065. *Limosilactobacillus fermentum,* also obligate heterofermentative LAB, was detected in NG-26064 and NG-26434 isolates at relative abundance of 0.06 and 0.19%, respectively. In addition, *Acinetobacter lwoffii* was identified only in NG-26064, with relative abundance of 0.04%.

Some substantial differences were observed between the results obtained with the QIAamp kit and both NucleoSpin and GENESpin kits. Genus *Weissella* was identified with a relative abundance of 36.31% with QIAamp, which was significantly higher than the percentage identified by both NucleoSpin (3.67%) and GENESpin (6.69%) kits (Fig. 2). On the contrary, genus *Lactobacillus* was identified at the lowest percentage (63.11%) in NG-26065 (QIAamp kit), compared with 82.28 and 70.08% in NG-26064 (NucleoSpin kit) and NG-26434 (GENESpin kit), respectively. *L. plantarum* was present at 31.24% in NG-26065 (QIAamp) isolate compared to 16% (16.15 and 16.28% respectively) of NucleoSpin and GENESpin isolates. However, *L. brevis* was identified at the lowest percentage in QIAamp isolate (6.21%) compared to 14.46 and 16.29% in NG-26064 (NucleoSpin kit) and NG-260434 (GENESpin kit) isolates, respectively. Therefore, similarity in the taxonomic composition of bacterial community in NG-26064 and NG-26434 isolates was identified in contrast to NG-26065 isolate, which differs mainly in the proportional distribution of the established ASVs. The results obtained with the QIAamp kit can be only partly explained by the significant removal of background DNA in the particular isolate. The relative abundance of background DNA was 0.48% in NG-26065, 12.74% in NG-26064 and 22.99% in NG-26434 isolate. Having in mind that the presence of a large amount of background DNA can negatively affect the 16S rRNA gene sequence analysis, it seems that the GENESpin kit is less suitable for microbiome profiling of sourdough. On the other hand, the 16S rRNA gene libraries prepared from metagenomic DNA extracted with the GENESpin kit had better diversity and evenness (Shannon and Simpson) indices (Table 3) than NG-26065 (QIAamp) isolate.

**Fig. 3:**
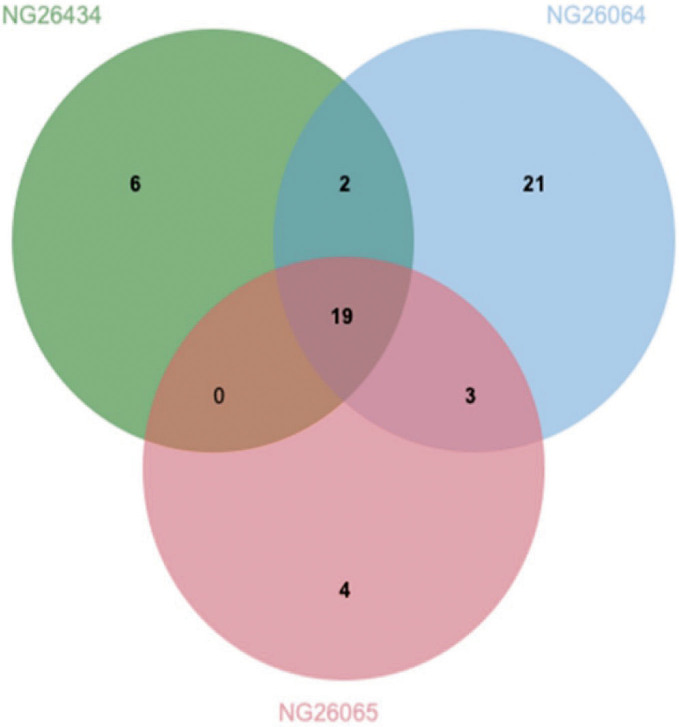
Venn diagram analysis of the common and unique amplicon sequence varians (ASVs) obtained by DNA extraction with different kits. Blue circle depicts ASVs found in isolate NG-26064 obtained from NucleoSpin Food kit (method 1), red circle depicts ASVs found in isolate NG-26065 obtained from QIAamp DNA Microbiome kit (method 2), and green circle represents ASVs found in isolate NG-26434 obtained from GENESpin Food kit (method 3)

In order to illustrate the information from the NGS analysis, a Venn diagram was used to depict how the results obtained from the three kits (the shared and unique ASVs) relate to each other against an overall ASVs dataset (Fig. 3). The three isolates shared only 19 of the total 98 recovered ASVs, indicating that the DNA extraction methods significantly influenced the analysis of microbiota community structure. Hence, the overlapping of 19.34% of ASVs showed that the different extraction methods captured different bacteria in the tested sourdough. The shared ASVs between NucleoSpin and QIAamp methods were 22.45%, between the NucleoSpin and GENESpin 21.43%, and between the GENESpin and QIAamp 19.34%. Other authors also reported that different DNA extraction methods affected the recovery of ASVs, hence the evaluation of microbiota of naturally fermented foods (*40*).

The data obtained from NGS analysis of the three isolates together provided insight into the microbiota composition of the investigated Bulgarian sourdough and the contribution of the established bacteria to the characteristics of the final product. It is well known that the metabolic activities of lactic acid bacteria improve the bread structure and prolong shelf life due to acidification and antimicrobial compound production (*44*). According to the data from the literature, in sourdough, *Lactobacillus* strains are more frequently found than *Leuconostoc* and *Weissella* species. Our results confirmed the dominance of *Lactobacillus* (63.11–82.28%) and subdominant presence of *Weissella* (3.67–36.31%), followed by *Leuconostoc* with significantly lower relative abundance (0.09–0.24%) in all three isolates of the investigated Bulgarian sourdough. Other authors also showed the important role of the representatives of the two genera *Lactobacillus* and *Weissella* in the fermentation processes of the sourdough (*45*). LAB found in sourdough mainly belong to genus *Lactobacillus* (*43*). In confirmation of our results, there are published data that *L. plantarum* and *L. brevis* are dominant LAB in the sourdough as well as that *L. plantarum* is generally codominant with obligate heterofermentative LAB, including *L. brevis* (*46*). Homofermentative and heterofermentative LAB perform different functions and contribute to the final product quality (*1*). Heterofermentative LAB mainly produce ethyl acetate with some alcohols and aldehydes, and homofermentative LAB produce diacetyl and other carbonyls. Among different metabolites produced by LAB, lactic and acetic acids are thought to be the main organic antifungal compounds (*46*). *L. plantarum* produces a high amount of acetic acid in sourdough as well as bacteriocins, which have an inhibiting effect against pathogens like *Bacillus subtilis* as well as antifungal activity (*43*). Preservative effect, which prolongs the shelf life of the products, was also reported for the well-adapted LAB *L. fermentum* due to its anti-mould activity (*47*). Furthermore, dough acidification has been shown to have significant effects on the quality characteristics of bread such as texture and volume. The lactic acid mainly produced by heterofermentative LAB may be responsible for more elastic gluten structure. The organic acids, lactic and acetic, are also responsible for creating different taste and odour in sourdough (*46*). *L. brevis* produced higher amounts of organic acid than *L. plantarum*. In addition, *L. plantarum* are known to produce a wide range of volatile compounds (*44*). *L. plantarum* and *L. brevis* have beneficial effects on bread organoleptic properties (volume, crumb texture, unique flavour) (*46*). Therefore, the synergetic effect of the LAB strains in the sourdough had a significant role in the improvement of volume, texture, staling rate and microbial shelf life. The heterofermentative cocci *Weissella* and *Leuconostoc,* also found in the investigated sourdough, can be important for growth association with lactobacilli (*1*). Recently, in a study comparing starters composed of different combinations of dominant bacteria, the best result was obtained with a culture containing *Lactiplantibacillus plantarum* 2MI8 and exopolysaccharide-producing *Weissella confusa/cibaria* 6PI3 strains (*48*).

The presence of *Acinetobacter lwoffii* in the investigated sourdough, found in a very low count only in NG-26064 isolate, could be explained by contamination (from the environment). *Acinetobacter lwoffii*, like other species from genus Acinetobacter, is common in marine fish and water microbiome and is considered as pathogen. *Acinetobacter lwoffii* was found in some traditionally fermented foods like traditional sour cream from Russia (*49*) as well as in vegetables like Chinese chive (*50*).

## CONCLUSIONS

In this work we analyzed the taxonomic composition of the bacterial community of a traditional sourdough originating from Southwestern Bulgaria using three commercial kits for DNA isolation. The 16S rRNA next-generation sequencing (NGS) data confirmed that the choice of DNA extraction protocol is a key factor in metagenomic analysis due to the variations generated in the recovery of amplicon sequence variants by different methods of DNA isolation. The data also revealed that each of the tested kits could be used for DNA isolation and estimation of the bacterial community of sourdough, although the best results in this study were achieved with the NucleoSpin kit. The diversity in the bacterial community profiles recovered by different methods emphasized the necessity of a selection and validation of a standard protocol for isolation of bacterial DNA for NGS analysis of fermented foods, in particular sourdough.
